# Reversible Protonic Doping in Poly(3,4-Ethylenedioxythiophene)

**DOI:** 10.3390/polym10101065

**Published:** 2018-09-25

**Authors:** Shuzhong He, Masakazu Mukaida, Kazuhiro Kirihara, Lingyun Lyu, Qingshuo Wei

**Affiliations:** 1 School of Pharmaceutical Sciences, Guizhou University, Guiyang 550025, China; pmc.szhe@gzu.edu.cn; 2 Nanomaterials Research Institute, Department of Materials and Chemistry, National Institute of Advanced Industrial Science and Technology (AIST), 1-1-1 Higashi, Tsukuba 305-8565, Japan; mskz.mukaida@aist.go.jp (M.M.); kz-kirihara@aist.go.jp (K.K.); 3 AIST-UTokyo Advanced Operando-Measurement Technology Open Innovation Laboratory (OPERANDO-OIL), National Institute of Advanced Industrial Science and Technology, 1-1-1 Higashi, Tsukuba 305-8565, Japan; jessica0107.lyu@aist.go.jp; 4 Precursory Research for Embryonic Science and Technology (PRESTO), Japan Science and Technology Agency, 4-1-8 Honcho, Kawaguchi 332-0012, Japan

**Keywords:** organic semiconductor, oxidative doping, protonic doping

## Abstract

In this study, poly(3,4-ethylenedioxythiophene), a benchmark-conducting polymer, was doped by protons. The doping and de-doping processes, using protonic acid and a base, were fully reversible. We predicted possible doping sites along the polymer chain using density functional theory (DFT) calculations. This study sheds potential light and understanding on the molecular design of highly conductive organic materials.

## 1. Introduction

Previous studies have successfully used conducting polymers, as active materials, in different electronic devices such as organic light-emitting diodes (OLEDs), organic solar cells (OSCs), thin film transistors (TFTs), thermoelectrics, batteries, and biosensors [1]. Among the different types of conducting polymers, poly(3,4-ethylenedioxythiophene) (PEDOT) has received the most attention, both academically and in practical applications [2]. It is widely applied as a hole-transporting layer in OLEDs and OSCs and as a source–drain–gate electrode for TFTs. Oxygen reduction catalysts also use PEDOT as a replacement for Pt in fuel cells [3]. More recently, PEDOT has been used as a thermoelectric material to directly convert heat into electricity [4,5,6,7,8,9]. The solution-processable PEDOT film was able to provide high electrical conduction (>4000 S/cm), and single-crystal PEDOT nanowires, synthesized under geometrically-confined conditions, provided electrical conduction as high as 8000 S/cm [10,11,12]. It is reasonable to believe that the highly conductive PEDOT films were doped at the high levels. However, doping mechanisms are still not well understood. This lack of understanding places limitations on the design of highly conductive organic materials, on further improvements to PEDOT system applications in various devices (e.g., optimization of the thermoelectric properties of PEDOT based materials), and on the understanding of how oxygen reduction mechanisms work in a wide pH range [3,13].

Previous studies have identified two different doping mechanisms in *p*-type conducting polymers [14,15]. The first is oxidative doping, a chemical oxidation reaction, based on a charge transfer from the host to the dopant. Conducting polymers doped with I_2_, FeCl_3_, SbCl_5_, and tetracyanoquinodimethane (TCNQ) are based on oxidative doping mechanisms [15]. The second is protonic doping, which has been studied in nitrogen-containing organic semiconductors (e.g., polyaniline) [16,17,18]. Protons interact at the nitrogen sites in these polymers, and the charge delocalizes within the polymer chains, providing electronic conductivity. PEDOT is known to be synthesized using Fe^3+^ or peroxodisulfate as an oxidant and has no nitrogen sites in it [19]. Therefore, in general, it is believed that PEDOT is oxidatively doped by oxidizing agents.

Using spectroscopic analysis to monitor the amount of oxidizing agents during polymerization, previous studies have reported that one positive charge exists for every three thiophene rings. The maximum carrier concentration via oxidation is approximately 3 × 10^20^ cm^−3^, assuming that the weight ratio between PEDOT and the dopant polystyrene sulfonate (PSS) is 1:2.5 [2]. Using the maximum carrier density, for a film with an electrical conductivity of 4000 S/cm, the calculated carrier mobility is greater than 80 cm^2^/Vs. This value was too high, even when compared with single-crystal organic semiconductors [20,21], and indicates that PEDOT carrier concentrations should be much larger than the value that is obtained when only considering oxidative doping. In other words, there is a possible presence of protonic doping in the PEDOT system. In this study, we show that there is protonic doping in PEDOT and that the doping/de-doping process is fully reversible in a solid state. Using density functional theory (DFT) calculations, we propose the possible doping sites in the polymer chains. This study is not only important for PEDOT applications but could also contribute toward the molecular design of highly conductive organic materials.

## 2. Experimental Section

### 2.1. Materials

PEDOT:PSS (Clevios PH1000) was purchased from H.C. Starck (Goslar, Germany). Sulfuric acid, potassium hydroxide and ethylene glycol were purchased from TCI Chemicals (Tokyo, Japan).

### 2.2. Film Preparation

An aqueous PEDOT:PSS solution containing 3% EG was used as the ink. Adding EG to the PEDOT:PSS solution (or treating the PEDOT/PSS film by EG) improved the crystallinity of PEDOT and the ordering of the PEDOT nanocrystals in the solid films [22]. The PEDOT/PSS film was first spin coated onto glass and annealed at 150 °C for 10 min in air. The film treatment was then carried out by immersing the film in different solutions for 5 min and then drying it in air at 150 °C for 5 min.

### 2.3. Characterization

The film thickness was measured using a surface profilometer (Surfcoder ET 200, Kosaka Laboratory Ltd., Tokyo, Japan). The conductivity was also cross-checked using a four-probe conductivity meter (MCP-T600, Mitsubishi Chemical Corporation, Tokyo, Japan). The ultraviolet–visible–near-infrared (UV–Vis–NIR) spectra were acquired using a UV–Vis–NIR spectrometer (Solidspec-3700, Shimadzu, Kyoto, Japan). The FTIR spectra were acquired using an ALPHA FTIR Spectrometer from Bruker, Yokohama, Japan. The Seebeck coefficient was measured using a laboratory-built apparatus, and the technique used to measure the Seebeck coefficient is reported in a previous study [23,24]. All calculations were performed using the Gaussian 09 Rev. E. 01 program.

## 3. Results and Discussion

**Figure 1:**
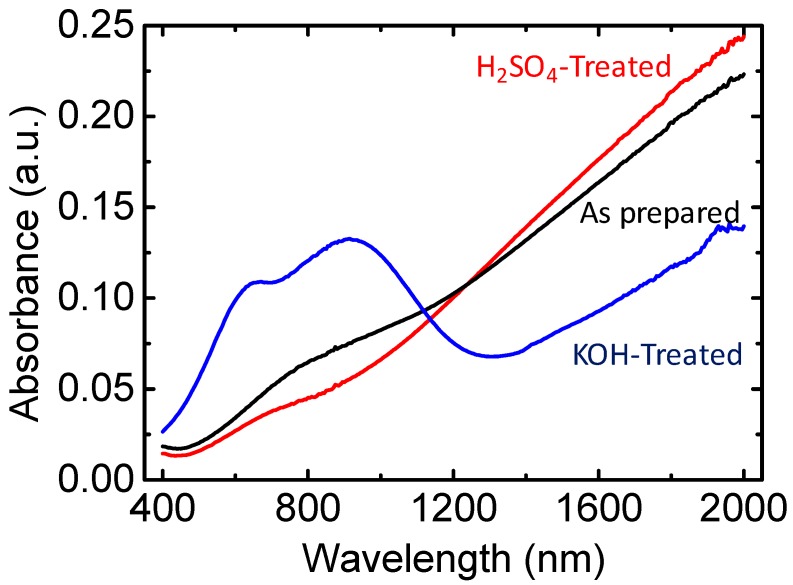
Absorption spectra of films synthesized from PEDOT/PSS (poly(3,4-ethylenedioxythiophene)/polystyrene sulfonate): H_2_SO_4_-treated PEDOT/PSS and KOH-treated PEDOT/PSS.

**Figure 2:**
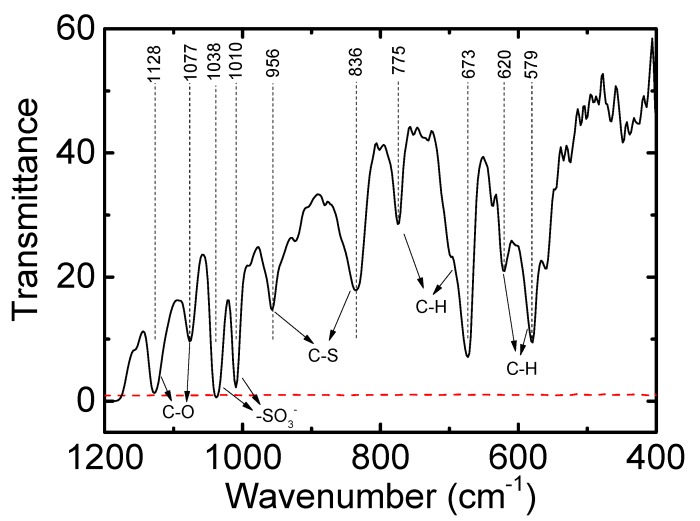
FTIR spectra of a free-standing PEDOT/PSS film before (red dotted line) and after (solid line) 1 M KOH treatment.

We focused on color changes in the PEDOT/PSS film during fabrication under varying conditions. Figure 1 displays the ultraviolet–visible–near-infrared (UV–Vis–NIR) spectra for a 50-nm PEDOT/PSS film. In this figure, the black line represents the as-prepared sample. After treatment in a solution of 1 M H_2_SO_4_, the film’s absorption increased at wavelengths larger than 1200 nm and decreased at wavelengths lower than 1200 nm, resulting in a more transparent film. However, when we treated the as-prepared PEDOT/PSS film in a solution of KOH we observed trends that were opposite to those for the H_2_SO_4_ treatment: The film’s absorption decreased at wavelengths higher than 1200 nm, polaron absorption occurred at approximately 950 nm, and neutral-state absorption at approximately 650 nm, resulting in a blue film. The free-standing PEDOT/PSS films treated with KOH became IR transparent, and the C=O, C–H, and –SO_3_^−^ vibrations were observable (Figure 2). This result suggested that the number of free carriers in the film increased after H_2_SO_4_ treatment and decreased after KOH treatment. Please note that the PEDOT film is much thinner on KBr substrates and is partly transparent in the IR range which makes the FTIR measurement on doped PEDOT/PSS films possible [25]. We could not observe other peaks such as the free SO^4−^ in our samples, which may relate to lower transmittance of our samples.

**Figure 3:**
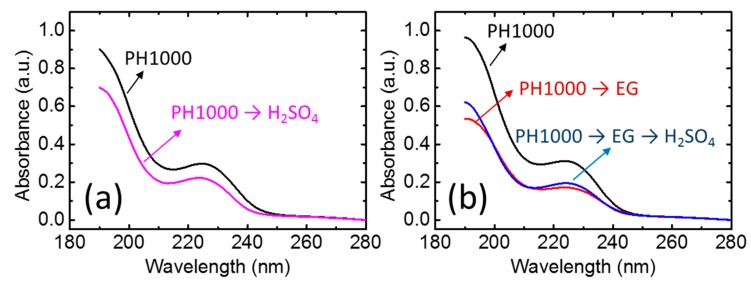
Absorption spectra of: (**a**) PEDOT/PSS films and H_2_SO_4_-treated PEDOT/PSS films; (**b**) PEDOT films, EG-treated PEDOT/PSS films and H_2_SO_4_–EG-treated PEDOT/PSS films.

It is well known that acid treatment can enhance the electrical conductivity of PEDOT [11,12,26,27]. This is mainly attributed to the removal of the insulating PSS^−^ and the morphological change of the film. In this study, we firstly compared the film absorption in the UV range of four samples: PEDOT/PSS without any additional treatment (PH1000), PEDOT/PSS treated with H_2_SO_4_ (PH1000 → H_2_SO_4_), PEDOT/PSS treated with EG (PH1000 → EG), and PEDOT/PSS treated with EG and H_2_SO_4_ (PH1000 → EG → H_2_SO_4_). This experiment was conducted using the same film with an alignment mark to avoid the effect of film thickness on the absorbance measurement. As shown in Figure 3a, after H_2_SO_4_ treatment, the absorption of PEDOT/PSS at 225 nm decreased suggesting the removal of the insulating PSS^−^, whereas, the electrical conductivity significantly increased to ca. 2000 S/cm (Table 1). A similar tendency was observed in the EG treated films. In Figure 3b, after EG treatment, the absorption of PEDOT/PSS decreased and the electrical conductivity increased to ca. 1000 S/cm. Interestingly, when we further treated this film using H_2_SO_4_, the absorption in the UV range did not show a significant change, suggesting the amount of PSS^−^ remained constant. On the other hand, the electrical conductivity further increased to ca. 2000 S/cm, which was almost identical to the film treated with H_2_SO_4_ (PH1000 → H_2_SO_4_). These results suggest that, besides the removal of the insulating PSS^−^, the treatment of H_2_SO_4_ also enhanced the electrical conductivity of the films.

Another possible mechanism of acid treatment is that the charge transportation is enhanced by the cations. Since PEDOT is a p-type semiconductor, the Coulombic interaction between PSS^−^ and moving carriers may be screened by a cation. To confirm this effect, we treated the films using Li_2_SO_4_, Na_2_SO_4,_ and K_2_SO_4_. As shown in Table 2, the electrical conductivity was not significantly enhanced, like it was by H_2_SO_4_, suggesting that protons played an important role in the electrical conductivity enhancement. As an assumption here, we believed that protons enhanced the carrier concentration in the PEDOT/PSS films which caused the electrical conductivity to increase.

**Figure 4:**
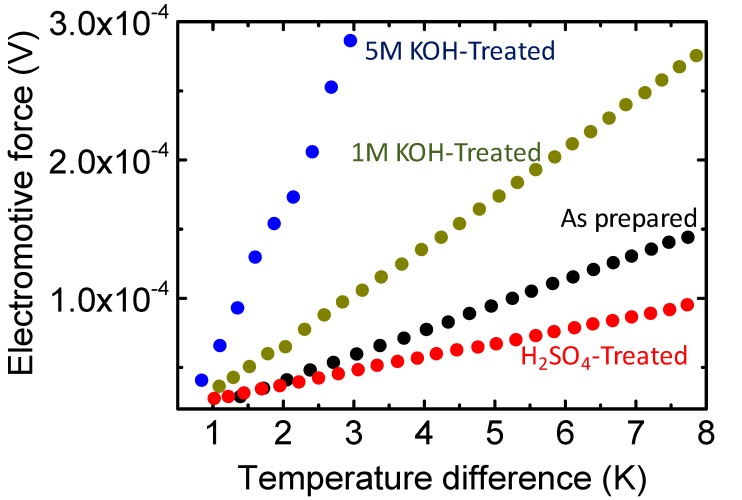
Seebeck coefficient measurements for PEDOT/PSS films treated under varying conditions.

Direct measurement of the PEDOT carrier concentration and mobility using the Hall effect is challenging because it is a disordered system [28]. To confirm carrier concentration changes during the KOH and H_2_SO_4_ treatments, we analyzed the thermopower of the films (i.e., the Seebeck coefficient). The Seebeck coefficient is sensitive to the film’s carrier density but less sensitive to the film’s morphology [29]. Recent studies have shown that the thermoelectric performance of PEDOT can be optimized using acid or base treatments, although these mechanisms are not clear [6,7,30]. Comparing the film’s Seebeck coefficient after each different treatment is a conclusive experiment to understand relative carrier number changes in the film. We measured the Seebeck coefficient using a home-made setup (details are reported in previous research). The PEDOT/PSS film Seebeck coefficients were measured for temperature differences between 1 and 8 K. The values of *S* were calculated from the slopes of the ∆*V* versus ∆*T* plots. As shown in Figure 4, the as-prepared PEDOT/PSS films (PH1000 with EG) yielded a Seebeck coefficient of 18 μV/K, which is close to those of the films reported in previous studies [29]. The electrical conductivity was ~1000 S/cm. After treatment with 1 M H_2_SO_4_, the Seebeck coefficient decreased to ~10 μV/K and the electrical conductivity increased to 2025 S/cm. This suggested that the number of holes in the PEDOT/PSS film increased after acid treatment. When treated with a 1 M KOH solution, we obtained an increased Seebeck coefficient of 35 μV/K for the film and a decreased electrical conductivity of 53 S/cm. With higher concentrations of KOH, we observed Seebeck coefficients as high as 142 μV/K with much lower electrical conductivity (i.e., smaller than 1 S/cm). This suggested that the carrier concentration significantly decreased after treatment with a base. The KOH treatment did not change the amount of PSS^−^ in the film, as shown in Supporting Information (Figure S1). This carrier concentration change trend is consistent with observations made using UV–Vis–NIR, as shown in Figure 1. Table 3 summarizes the film’s electrical conductivity and Seebeck coefficients using KOH and H_2_SO_4_ treatments.

**Figure 5:**
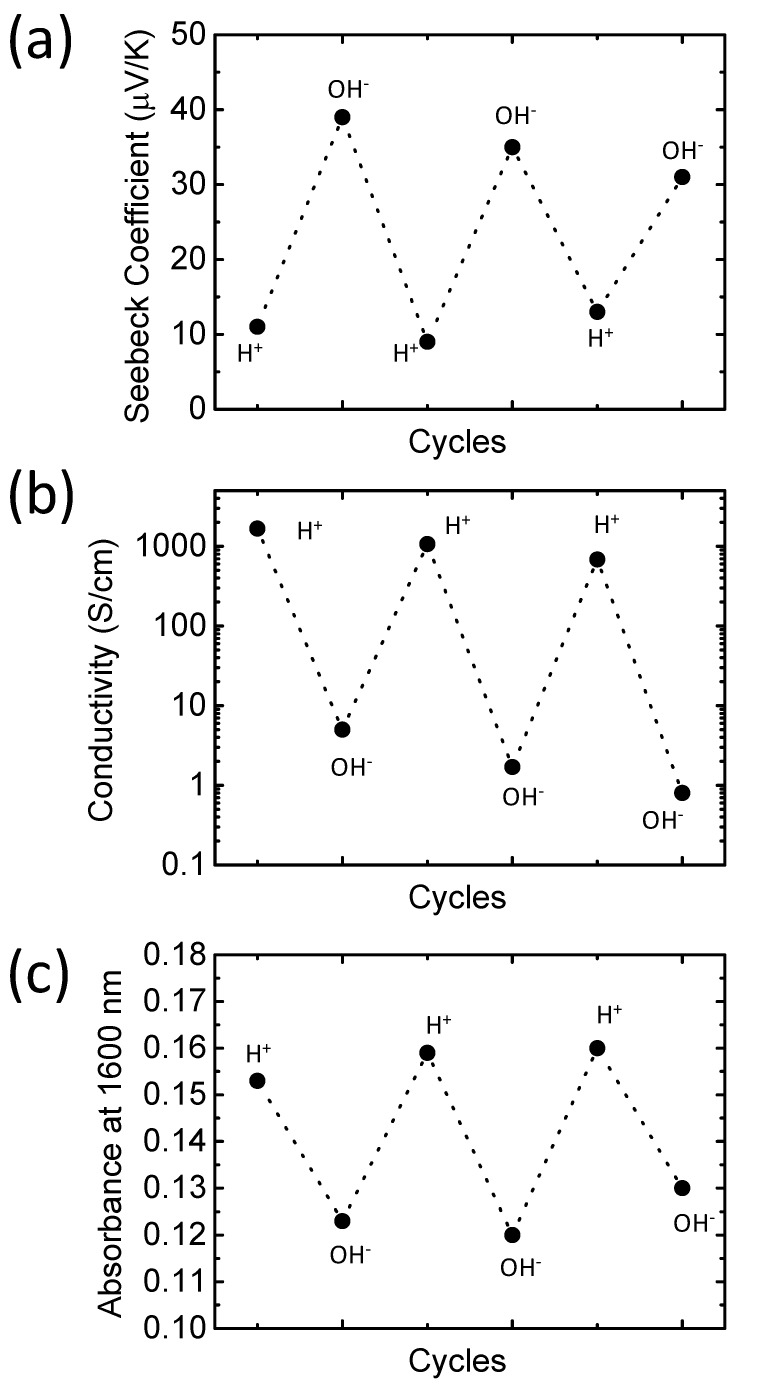
Reversibility of doping and de-doping performed on a poly(3,4-ethylenedioxythiophene)/polystyrene sulfonate (PEDOT/PSS) film with H_2_SO_4_ and KOH treatments. (**a**) Seebeck coefficient, (**b**) electrical conductivity, and (**c**) film absorbance at 1600 nm.

If protons interact with polymer chains similar to polyaniline, the doping/de-doping process using H^+^ and OH^−^ should be reversible with the same type of film that was employed herein [16]. To confirm protonic doping reversibility for PEDOT/PSS films, we monitored film absorption at 1600 nm, the Seebeck coefficient, and the electrical conductivity during H_2_SO_4_/KOH treatment cycles. As shown in Figure 5a, the Seebeck coefficient decreased during the H_2_SO_4_ treatment, and the electrical conductivity (Figure 5b) and absorption at wavelengths greater than 1200 nm (Figure 5c) increased. On the contrary, when we treated the film with KOH, the Seebeck coefficient increased, whereas electrical conductivity and absorption at wavelengths greater than 1200 nm decreased. It is worth noting that the doping and de-doping processes are fully reversible when using the same film. This suggests that protons are capable of reacting with polymer chains even when in a solid state.

**Figure 6:**
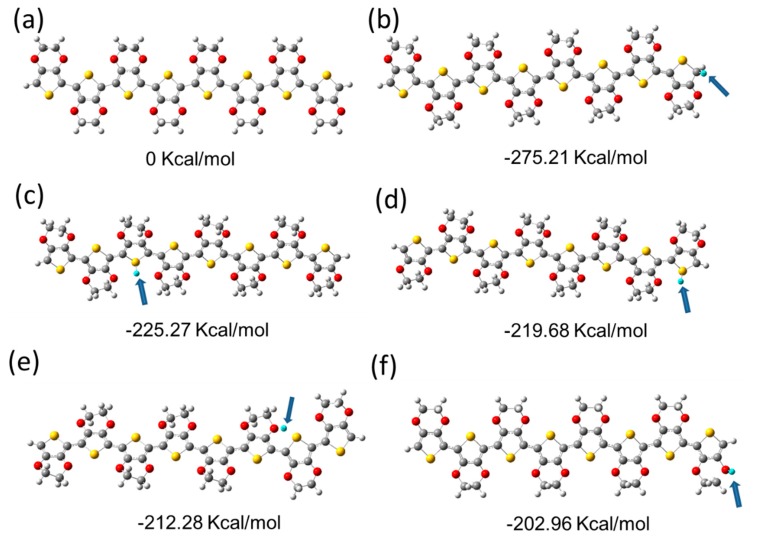
Ground-state geometry optimizations of: (**a**) octa-EDOT, adducts with H^+^ on (**b**) carbon of the end-thiophene, (**c**) sulfur of the middle thiophene, (**d**) sulfur of the end-thiophene, (**e**) oxygen of the middle thiophene, and (**f**) oxygen of the end-thiophene. The H^+^ atoms are light-blue in color and are indicated by blue arrows. Optimized structures were calculated using density functional theory (DFT) at the B3LYP/6-31G(d) theory level. Relative energies are shown under each diagram.

We experimentally confirmed that the proton concentration affects the PEDOT carrier concentration. However, it is not clear how to obtain the doping sites from the experiment because PEDOT is not soluble in the solvent after film formation. DFT methods were used to understand proton–PEDOT integration mechanisms [31]. First, we optimized octa-EDOT at the B3LYP/6-31G(d) theory level (The number of repeating units in PEDOT is between 6 to 10; we chose octamater for our calculations), as shown in Figure 6a. We subsequently optimized adducts with protons independently bound to C– (Figure 6b), S– (Figure 6c,d), and O– (Figure 6e,f) atoms in the octa-EDOT compound. The relative energies determined from the optimized structures showed that, compared with adduct formation at other positions, the adduct formation at the α-position of thiophene is the most favorable at a minimum of ~50 Kcal/mol. This is consistent with current knowledge of the reactivity of thiophene as the α-position is known to be active and the HOMO electron density at the α-position is known to be high [32,33]. It is important to note that there are not a small number of α-positions on the end-thiophene, because the PEDOT molecular weight is low, which suggests that only protonation at the end-thiophene α-position could provide a high carrier density. Adducts with protons bound to the S-atom in the middle thiophene rings are a second favorable position (Figure 6c). Recent studies have shown that polythiophene with a much larger molecular weight (i.e., a small amount of end-thiophene) is also able to be doped by protons, providing high electrical conductivity [34,35]. The probability of adduction formation with protons bound to O–atoms is unlikely. There are significant structural changes to adducts with protons bound to O–atoms (e.g., the proton is far from oligothiophene or a thiophene ring opens, as shown in Figure 6e,f). In the π-conjugated backbone, protons bound to carbon have sp^3^ bonds which break the conjugation [36]. It is important to point out that protonic acid is not always used as a starting material during PEDOT synthesis. However, the polymerization process generates protonic acid.

Finally, it is important to point out that the reversible change of electrical conductivity [37] and Seebeck coefficient [7] during acid/base treatment has been reported by Ouyang’s group [7] and Okuzaki’s group [37], although there are differences in the proposed mechanisms. Okuzaki’s group suggested that OH^−^ treatment of PEDOT/PSS films disrupts π–π stacking of PEDOT nanocrystals and therefore the electrical conductivity decreases. The addition of H_2_SO_4_ then restores this structure, and hence the conductivity increased again. Ouyang’s group suggested that there is a strong Coulombic attraction between OH^−^ and PEDOT^+^, so the charge carriers become more localized after OH^−^ treatment. Experimental results support both mechanisms. Their reports also reflected the fact that doping in PEDOT/PSS is still not fully understood, and thus, we trust that the protonic doping of PEDOT proposed in this paper is not in contradiction to the proposed mechanisms by pioneering groups. It is known that the carrier localization is strongly dependent on carrier concentration in the doped organic semiconductors [38]. A decrease in carrier concentration after OH^−^ treatment could also make the remaining charge carriers more localized due to Coulombic interaction.

## 4. Conclusions

In conclusion, we showed that: Protons dope PEDOT; protonic doping/de-doping is fully reversible; adducts with protons bound at the end-thiophene α-position are most favorable, based on DFT calculations; adducts with protons bound to the S–atom in the middle thiophene ring are also possible; and the use of protonated EDOT moieties, as an end group to prepare organic conductors, is a promising approach toward synthesizing highly conductive organic materials.

## Figures and Tables

**Table 1:** Summary of the electrical conductivity for different samples.

Sample Name	Electrical Conductivity (S/cm)
PH1000	2.5 ± 1.2
PH1000 → H_2_SO_4_	2118 ± 102
PH1000 → EG	971 ± 166
PH1000 → EG → H_2_SO_4_	1968 ± 150

**Table 2:** Summary of the electrical conductivity for different samples.

Sample Name	Electrical Conductivity (S/cm)
PH1000 → Li_2_SO_4_	3.7 ± 0.5
PH1000 → EG → Li_2_SO_4_	950 ± 120
PH1000 → Na_2_SO_4_	2.4 ± 0.3
PH1000 → EG → Na_2_SO_4_	932 ± 77
PH1000 → K_2_SO_4_	2.4 ± 0.1
PH1000 → EG → K_2_SO_4_	998 ± 167

**Table 3:** Summary of the electrical conductivity and Seebeck coefficients for different samples.

Sample Name	Electrical Conductivity (S/cm)	Seebeck Coefficient (μV/K)
As prepared (PH1000–EG)	1012	18
1 M H_2_SO_4_ treated	2025	10
1 M KOH treated	53	35
5 M KOH treated	0.9	142

## Supplementary Materials

The following are available online at http://www.mdpi.com/2073-4360/10/10/1065/s1.
