# Supplementary material for: Reversible Protonic Doping in Poly(3,4-Ethylenedioxythiophene)
Source: Polymers (Basel). 2018 Sep 25;10(10):1065. doi: 10.3390/polym10101065 (PMC6404222; doi:10.3390/polym10101065)
Supplement: Supplementary file 1 [file polymers-10-01065-s001.pdf]

Supporting Information

Reversible Protonic Doping in Poly(3,4-ethylenedioxythiophene)

Shuzhong He [1], Masakazu Mukaida [2,3], Kazuhiro Kirihara [2], Lingyun Lyu[3], Qingshuo Wei [2,3,4,*]

[1] School of Pharmaceutical Sciences, Guizhou University, Guiyang, Guizhou 550025, China; pmc.szhe@gzu.edu.cn

[2] Nanomaterials Research Institute, Department of Materials and Chemistry, National Institute of Advanced Industrial Science and Technology (AIST), 1-1-1 Higashi, Tsukuba, Ibaraki 305-8565, Japan; mskz.mukaida@aist.go.jp (M.M.); kz-kirihara@aist.go.jp (K.K.)

[3] AIST-UTokyo Advanced Operando-Measurement Technology Open Innovation Laboratory (OPERANDO-OIL), National Institute of Advanced Industrial Science and Technology, 1-1-1 Higashi, Tsukuba, Ibaraki 305-8565 Japan;
jessica0107.lyu@aist.go.jp (L.L.)

[4] Precursory Research for Embryonic Science and Technology (PRESTO), Japan Science and Technology Agency, 4-1-8 Honcho, Kawaguchi, Saitama 332-0012, Japan

* Correspondence: qingshuo.wei@aist.go.jp; Tel.: +81-29-861-3385

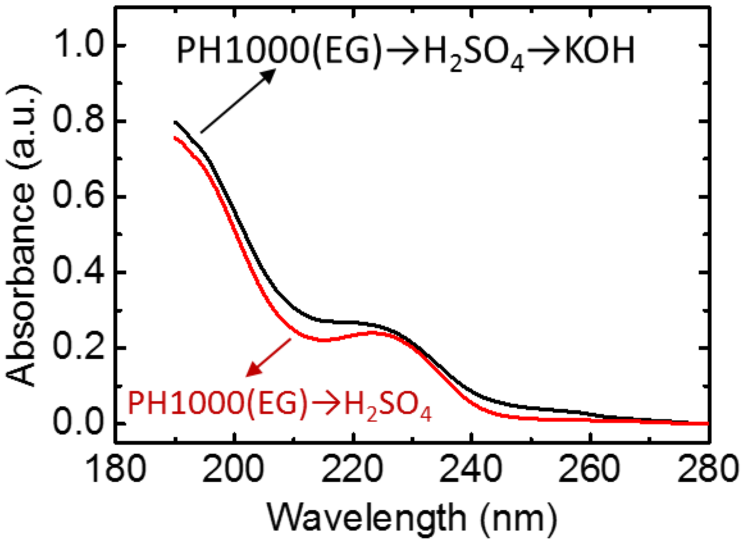

Figure S1. Absorption spectra of a H_2SO_4-treated PEDOT/PSS film and a KOH-H_2SO_4-treated PEDOT/PSS film.
